# Application of tangent-arc technology for deep inspiration breath-hold radiotherapy in left-sided breast cancer

**DOI:** 10.3389/fonc.2023.1145332

**Published:** 2023-08-18

**Authors:** Yucheng Li, Wenming Zhan, Yongshi Jia, Hanchu Xiong, Baihua Lin, Qiang Li, Huaxin Liu, Lingyun Qiu, Yinghao Zhang, Jieni Ding, Chao Fu, Weijun Chen

**Affiliations:** ^1^ Cancer Center, Department of Radiation Oncology, Zhejiang Provincial People’s Hospital, Affiliated People’s Hospital, Hangzhou Medical College, Hangzhou, Zhejiang, China; ^2^ Department of Tumor Radiochemotherapy, Second Affiliated Hospital of Wenzhou Medical University, Wenzhou, Zhejiang, China

**Keywords:** deep inspiration breath-hold, left breast cancer, dosimetry, organ of risk, continuous semi-arc, tangent-arc

## Abstract

**Objective:**

To explore the advantages of dosimetry and the treatment efficiency of tangent-arc technology in deep inspiration breath-hold radiotherapy for breast cancer.

**Methods:**

Forty patients with left-sided breast cancer who were treated in our hospital from May 2020 to June 2021 were randomly selected and divided into two groups. The first group’s plan was a continuous semi-arc that started at 145° ( ± 5°) and stopped at 325° ( ± 5°). The other group’s plan, defined as the tangent-arc plan, had two arcs: the first arc started at 145° ( ± 5°) and stopped at 85° ( ± 5°), and the second arc started at 25° ( ± 5°) and stopped at 325° ( ± 5°). We compared the target dose, dose in organs at risk (OARs), and treatment time between the two groups.

**Results:**

The target dose was similar between the continuous semiarc and tangent-arc groups. The V_5_ of the right lung was significantly different between the two groups (Dif 5.52, 95% confidence interval 1.92-9.13, *t*=3.10, *P*=0.004), with the patients in the continuous semi-arc and tangent-arc groups having lung V_5_ values of (9.16 ± 1.62)%, and (3.64 ± 0.73)%, respectively. The maximum dose to the spinal cord was (1835.88 ± 222.17) cGy in the continuous semi-arc group and (599.42 ± 153.91) cGy in the tangent-arc group, yielding a significant difference between the two groups (Dif 1236.46, 95% confidence interval 689.32-1783.6, *t*=4.57, *P*<0.001). The treatment times was (311.70 ± 60.45) s for patients in the continuous semi-arc group and (254.66 ± 40.73) s for patients in the tangent-arc group, and there was a significant difference in the mean number of treatment times between the two groups (Dif 57.04, 95% confidence interval 24.05-90.03, *t*=3.5, *P*=0.001).

**Conclusion:**

Both the continuous semi-arc and tangent-arc plans met the clinical prescription dose requirements. The OARs received less radiation with the tangent-arc plan than the continuous semi-arc plan, especially for the lung (measured as V_5_) and the spinal cord (measured as the maximum dose). Tangent-arc plan took significantly less time than the continuous semi-arc, which can greatly improve treatment efficiency. Therefore, tangent-arc plans are superior continuous semi-arc plans for all cases.

## Introduction

In women, breast cancer is the most common malignant tumor and has the highest mortality and morbidity among all malignant tumors worldwide (1–4). Recently, radiation therapy coupled with breast-conserving surgery has become the standard treatment for many patients with breast cancer (5, 6). For patients with breast cancer on the left side, the radiation dose to the heart should be taken into account during radiation therapy because the tumor is relatively close to the heart. Although no studies have demonstrated that the minimum exposure dose causes radiation-induced cardiac injury, increased cardiac doses are associated with increased rates of cardiac and coronary events. Furthermore, cardiac damage is correlated with the mean cardiac dose, with an increase of 4%-16% in the rate of acute coronary events per 1 Gy (7–11). To reduce the dose to organs at risk (OARs) as much as possible, some scholars have proposed new improvements in imaging techniques and treatment planning systems and have introduced new irradiation techniques, such as deep inspiration breath hold (DIBH) and respiratory gating (RG) (12–16). The main techniques used in breast cancer radiotherapy are three*-*dimensional conformal radiation therapy (3D-CRT), intensity modulated radiation therapy (IMRT), and volumetric modulated arc therapy (VMAT). Compared to 3D-CRT, both IMRT and VMAT can improve the target volume’s conformity index (CI) and homogeneity index (HI) while reducing the dose to OARs (17–19). The difference between IMRT and VMAT is that, when treating patients, the IMRT gantry has a fixed angle during irradiation, whereas in VMAT, the gantry rotates while the beam is on. Therefore, VMAT technology can increase the CI of the target. In recent years, the application of VMAT combined with DIBH technology has further reduced the dose of OARs (20–22). The focus of medical physicists is the optimization of treatment efficiency and design of the X-ray angle in the radiotherapy plan such that the dose to the OARs can be reduced as much as possible while ensuring that the target volume receives a sufficient dose.

This study aims to explore a new tangent-arc irradiation technique based on DIBH. It is expected that this technique will allow patients with left-sided breast cancer to receive adequate doses of radiotherapy in the target region while further reducing the dose of OARs, especially the heart, lungs, and other organs that affect the quality of life of patients. It is also expected to reduce patients’ DIBH time which can effectively improve the efficiency of treatment time while improving patient cooperation. Thus, a high-quality and efficient plan design scheme is provided for patients with left breast cancer using the DIBH technique.

## Methods and materials

### Patient selection

**Figure 1:**
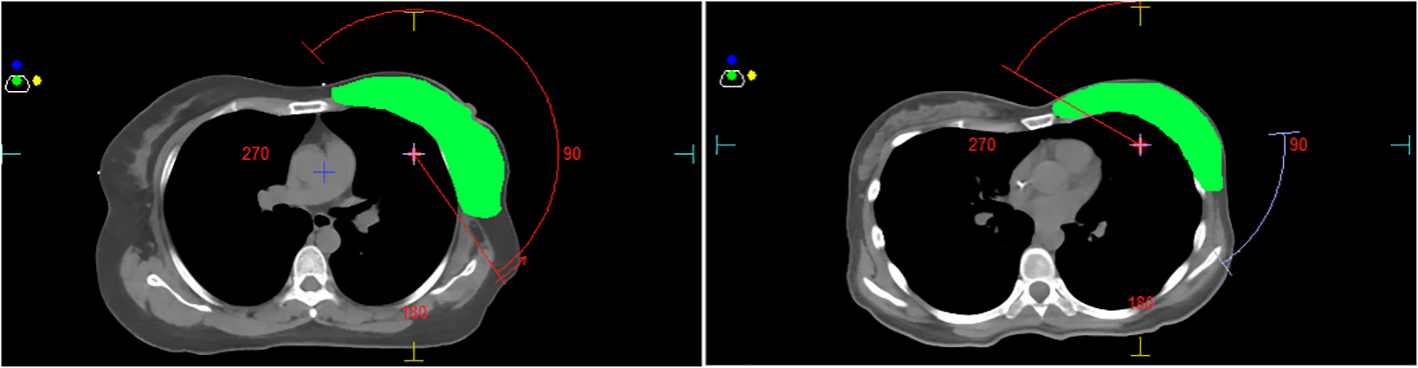
(The left is the continuous semi-arc, the right is the tangent-arc, and green represents PTV).

Forty patients with left-sided breast cancer who were treated in our hospital from May 2020 to June 2021 were randomly enrolled in this study and divided into two groups, one continuous semi-arc plan group and the other tangent-arc plan group. The continuous semi-arc plan had only one arc that rotated counterclockwise from 145° ( ± 5°) to 325° ( ± 5°). The tangent-arc plan had two arcs: the first arc rotated counterclockwise with a start angle of 145° ( ± 5°) and a stop angle of 85° ( ± 5°), and the second arc rotated counterclockwise from 25 ( ± 5)°to 325° ( ± 5°). The angles of the two plans are shown in **Figure 1**. Among them, the mean age of the 20 patients treated with continuous semi-arc technology was 47.1 (range 33-58) years, and the mean age of the 20 patients treated with tangent-arc technology was 45.7 (range 29-60) years. The inclusion criteria were left-sided breast cancer, no contraindications to radiotherapy, KPS> 70, age younger than 60 years old, ability to fully understand the process of DIBH, and ability to breath-hold for more than 30 s. All patients completed simulated positioning and surface-guided radiation therapy (SGRT) using Catalyst Systems v5.4.2 SP3 (C-RAD Positioning AB, Uppsala, Sweden) with DIBH to reduce localization uncertainty during treatment delivery. The exclusion criteria were a breath-holding time of fewer than 30 s, communication disorders, and other underlying diseases affecting radiotherapy.

### CT simulation positioning, target contour, planning design

All patients were laid in a supine position with both arms fully abducted and externally rotated on a vacuum cushion on the all-in-one board. Treatment planning CT scans at 5-mm intervals from the ear to 2 cm below the diaphragm were obtained for each patient with a CT simulator (Discovery CT590, GE, Wisconsin, USA). The target and OARs of this study were delineated following the Radiation Therapy Oncology Group (RTOG) and the International Commission Radiological Units (ICRU) (23, 24). The two groups of patients were treated with continuous semi-arc technology and tangent-arc technology. Both plans were generated using the MonacoV5.11 (Elekta AB, Stockholm, Sweden) three-dimensional treatment planning system by the same senior medical physicist. The “Dual Arc” function provided by the treatment planning system was used to generate clockwise and counterclockwise dual arcs for each plan. The doses were normalized such that the dose to 95% of the planning target volume (PTV) was the same for all plans.

### Dose evaluation

All plans were compared and evaluated. The near maximum dose covering 2% of the PTV (D_2%_), near minimum dose covering 98% of the PTV (D_98%_), and mean dose (D_mean_) to the PTV was determined. The volume of the left lung receiving dose greater than 5, 20, and 30 Gy (V_5_, V_20_, and V_30_, respectively) and the D_mean_ of the left lung were considered as well as the V_5_ and D_mean_ of the right lung, D_mean_ of the heart and left ventricle, maximum dose (D_max_) of the spinal cord, beam-on time, CI and HI. The CI was calculated from the formula: CI= (TV_95_/TV) × (TV_95_/V_95_), where V_95_ is the target volume receiving 95% of the prescription dose, TV is the target volume, and V_95_ is the volume receiving 95% of the prescription dose. HI was calculated according to HI=(D_5%_)/(D_95%_) where D_5%_ and D_95%_ represent doses received by 5% and 95% of PTV, respectively). The closer the CI and HI values are to 1, the better the quality of the plan. The treatment time of all patients was recorded by the catalyst software.

### Statistical analysis

All patient data were statistically analyzed using SPSS software (version 20, SPSS Inc., Chicago, IL, USA). The independent sample t-test was used to analyze parameters with homogeneous variance and normal distribution; otherwise, the nonparametric Wilcoxon signed-rank test was used. Data with a normal distribution are expressed as x ± s and were analyzed with the independent sample t-test, while those with a nonnormal distribution are presented as M (Q1, Q3) and were analyzed with the Mann-Whitney U test. A value of ***P*** < 0.05 was considered statistically significant.

## Results

Details of the dosimetry, treatment time, and beam-on-time comparisons are presented in **Tables 1** and **2**. The dose constraints were defined for OARs as follows: left lung: V_5_<50%, V_20_< 26%, V_30_< 20%; right lung: V_5_<12%; heart: D_mean_<7 Gy; left ventricle: D_mean_<7 Gy; spinal cord: D_max_< 40 Gy. The right lung V_5_ of the patients in the continuous semi-arc group and the tangent-arc group were (9.16 ± 1.62)% and (3.64 ± 0.73)%, respectively, with a significant difference between the two groups (Dif 5.52, 95% confidence interval 1.92-9.13, *t*=3.10, *P*=0.004). The maximum dose in the spinal cord was (1835.88 ± 222.17) cGy in the continuous semi-arc group and (599.42 ± 153.91) cGy in the tangent-arc group, and there was a significant difference between the two groups (Dif 1236.46, 95% confidence interval 689.32-1783.6, *t*=4.57, *P*<0.001). The treatment time was (311.70 ± 60.45) s for patients in the continuous semi-arc group and (254.66 ± 40.73) s for patients in the tangent-arc group, with a significant difference between the two groups (Dif 57.04, 95% confidence interval 24.05-90.03, *t*=3.5, *P*=0.001).

**Table 1:** Comparison of parameters between continuous semi-arc and tangent-arc ( $\bar{\chi }$ ± s).

parameters	continuous semi-arc	tangent-arc	Dif&95%confidence interval	*t*	*P*
PTVD_2%_(cGy)	5840.74 ± 470.47	5495.91 ± 704.10	344.84(-38.49-728.16)	1.82	0.076
PTVD_98%_(cGy)	4823.06 ± 185.46	4687.95 ± 336.43	135.11(-38.79-309.01)	1.57	0.124
CI	0.80 ± 0.06	0.81 ± 0.06	-0.01(0.05-0.02)	0.72	0.478
HI	1.18 ± 0.09	1.14 ± 0.08	0.04(-0.02-0.1)	1.42	0.163
L-Lung V_5_(%)	44.67 ± 6.03	42.28 ± 5.61	2.38(-1.35-6.11)	1.29	0.204
L-Lung V_20_(%)	20.25 ± 3.93	19.91 ± 3.89	0.09(-2.5-2.67)	0.273	0.786
L-Lung V_30_(%)	13.81 ± 3.62	14.21 ± 3.69	-0.41(-2.75-1.93)	-0.35	0.726
R-Lung V_5_(%)	9.16 ± 7.26	3.64 ± 3.28	5.52(1.92-9.13)	3.10	0.004
Heart D_mean_(cGy)	377.66 ± 73.89	379.92 ± 108.27	-2.27(-61.6-57.07)	-0.08	0.939
Left-ventricle D_mean_(cGy)	354.66 ± 89.61	389.31 ± 131.24	-34.64(-114.86-45.57)	-0.88	0.386
Spinal Cord D_max_(cGy)	1835.88 ± 993.57	599.42 ± 688.32	1236.46(689.32-1783.6)	4.57	0.000
Treatment time(s)	311.70 ± 60.45	254.66 ± 40.73	57.04(24.05-90.03)	3.50	0.001

**Table 2:** Comparison of the beam-on-time between continuous semi-arc and tangent-arc plans with different field angles.

plan	continuous semi-arc	tangent-arc		
degree	145°( ± 5°)~325°( ± 5°)	145°( ± 5°)~85°( ± 5°)	25°( ± 5°)~325°( ± 5°)	Total range time
Time(s)	82~130	41~57	38~60	81~110

## Discussion

Recently, with developments in radiotherapy physics and computing technologies, VMAT has become one of the mainstream technologies of radiotherapy. In particular, VMAT combined with DIBH can greatly reduce the dose of OARs while ensuring a sufficient dose to the target (20–22). Currently, the 5-year survival rate for stage I breast cancer is >85% worldwide and the majority of breast cancer patients can be cured with a combination of chemotherapy and radiotherapy (25–27). However, to our knowledge, there is no evidence proving that the minimum dose does not cause radiation-induced heart and lung injuries. Therefore, to improve the patient’s quality of life, medical physicists ensure that normal tissues are treated at as low a dose as possible while maintaining adequate target coverage. Comparing the time and dosimetry of two different VMAT techniques, This study showed that the tangent-arc technique was shown to reduce the dose to OARs and the treatment time compared to the continuous semi-arc plan.

The analysis showed that the maximum and minimum doses to the PTV increased by 5.9% and 2.8%, respectively, in the continuous semi-arc group compared with the tangent-arc group, but these increases were not statistically significant. The CI and HI of the two plans were also not significantly different. The reason for this lack of statistical difference may be that the dose of the target area was normalized to 95% for both the continuous semi-arc and tangent-arc plan designs.

Related studies have shown that V_5_, V_20,_ and V_30_ of the lung, especially V_20_, play important roles in radiation-induced pulmonary injury and fibrosis. When lung V_20_>20%, the probability of radiation pneumonitis is 28.4%, and when V_20_ ≤ 20%, the incidence of radiation pneumonitis is 12.5% (28–31). Here, the average V_20_ of the left lung of the continuous semi-arc plan was 20.25%, and that of the tangent-arc plan was 19.91%. Therefore, the tangent-arc plan may reduce the incidence of radiation pneumonitis. Additionally, the low-dose volume effect of the bilateral lung must be taken seriously in the clinical practice of breast cancer radiotherapy. Novakova-Jiresova et al. (32) conducted radiation-induced lung injury animal experiments, and showed that animals receiving low-dose and large-volume irradiation showed had greater lung function damage. John et al. (33) believed that larger lung volumes receiving low-dose irradiation would cause more severe radiation-induced lung damage. With the development of radiotherapy technology, the long-term survival rate of breast cancer has improved significantly. Some scholars have shown that low-dose radiation increases the risk and toxicity of secondary cancer (34, 35). Our results showed that the mean V_5_ value of the left and right lungs was reduced by approximately 5.4% and 60.26%, respectively, in the tangent-arc group compared with the continuous semi-arc group. Therefore, the V_5_ lung benefited from the use of tangent-arc (**Table 1**).

The results of this study showed that the mean cardiac doses of 377.66 cGy (continuous semi-arc group) and 379.92 cGy (tangent-arc group) in patients with breast cancer were lower than the 403 cGy value reported by Karpf et al. (36). The difference may result from the sample size and the volumes of the tumors. The author believe that this small difference would not affect the clinical benefit. Regarding the spinal cord, the maximum dose in the continuous semi-arc plan was approximately three times that of the tangent arc plan, possibly because the tangent-arc plan does not contribute any dosage to the spinal cord at 85°~25°, which is exactly the direction of vertical irradiation of the spinal cord, causing the spinal cord dose to drop significantly. Although the spinal cord doses of the two plans met the clinical dose requirement, the tangent-arc technique is more in line with the principle of being as low as reasonably achievable (37, 38).

The tangent-arc plan had shorter treatment time and X-ray beam-on time than the continuous semi-arc plan, and that the patient’s breath-hold interval was an important factor in the efficiency of treatment in the delivery treatment process. During the CT simulation, the patient must hold breath longer than 30 s. Then, in the continuous semi-arc plan, the beam-on time is 82~130 s, during which the patients can suffer from too little rest time which restricts their breathing, ultimately affecting the efficiency of treatment. However, in the tangent-arc plan, two small arcs are designed, with respective beam-on time of each arc is 41~57s and 38~60s, so the patient can complete each therapeutic arc in 1-2 breath-hold cycles. During the gantry rotation of the LINAC in between the two treatment fields, all patients were able to rest enough to maintain a stable breath-hold during the subsequent treatment field.

## Conclusions

Both continuous semi-arc and tangent-arc plans met the clinical prescription dose requirements. After comparing the radiation dose to OARs and the treatment time of patients, we believe that when left-sided breast cancer patients are treated with VMAT radiotherapy combined with DIBH, tangent-arc plans can be more effective. Tangent-arc plans can reduce the radiation dose to the patient’s OARs, such as the lung and spinal cord, and the treatment time can be faster. Therefore, the plan quality is superior for tangent-arc plans compared to continuous semi-arc plans for all cases. A limitation of this study is that there was no discussion of patient staging. The authors will further explore the advantages and disadvantages of using the two technical schemes in different stages.

## Data availability statement

The raw data supporting the conclusions of this article will be made available by the authors, without undue reservation.

## Ethics statement

The ethics institutional review board of Zhejiang Provincial People’s Hospital (Hangzhou, China) approved this study (project approval number QT2023020) and waived informed consent for this retrospective study.

## Author contributions

Manuscript drafting, editing, and statistical analysis: YL. Design, supervision, data interpretation, and critical review: WC. Patient surveillance and data acquisition:WZ, YJ, HX, CF, BL, and LQ. Literature search: QL, HL, and YZ, JD. All authors read and approved the final manuscript. All authors contributed to the article.
